# Relationship between efficacy and common metabolic parameters in first-treatment drug-naïve patients with early non-response schizophrenia: a retrospective study

**DOI:** 10.1186/s12991-023-00436-3

**Published:** 2023-02-17

**Authors:** Junhong Zhu, Jiajia Wu, Xuebing Liu, Jun Ma

**Affiliations:** 1 Department of Psychiatry, Wuhan Mental Health Center, No. 89, Gongnongbing Road, Jiang’an District, Wuhan, Hubei China; 2 Wuhan Hospital for Psychotherapy, No. 89, Gongnongbing Road, Jiang’an District, Wuhan, Hubei China

**Keywords:** Schizophrenia, Early non-response, Metabolic parameters, First-treatment, Drug-naïve

## Abstract

**Background:**

Comorbid metabolic disorders in patients with schizophrenia are very common. Patients with schizophrenia who respond to therapy early are often strongly predictive of better treatment outcomes. However, the differences in short-term metabolic markers between early responders and early non-responders in schizophrenia are unclear.

**Methods:**

143 first-treatment drug-naïve schizophrenia patients were included in this study and were given a single antipsychotic medication for 6 weeks after admission. After 2 weeks, the sample was divided into an early response group and an early non-response group based on psychopathological changes. For the study endpoints, we depicted the change curves of psychopathology in both subgroups and compared the differences between the two groups in terms of remission rates and multiple metabolic parameters.

**Results:**

The early non-response had 73 cases (51.05%) in the 2nd week. In the 6th week, the remission rate was significantly higher in the early response group than in the early non-response group (30,42.86% vs. 8,10.96%); the body weight, body mass index, blood creatinine, blood uric acid, total cholesterol, triglyceride, low-density lipoprotein, fasting blood glucose, and prolactin of the enrolled samples were significantly increased, and high-density lipoprotein was significantly decreased. ANOVAs revealed a significant effect of treatment time on abdominal circumference, blood uric acid, total cholesterol, triglyceride, high-density lipoprotein, low-density lipoprotein, fasting blood glucose and prolactin, and a significant negative effect of early non-response to treatment on abdominal circumference, blood creatinine, triglyceride, fasting blood glucose.

**Conclusions:**

Schizophrenia patients with early non-response had lower rates of short-term remission and more extensive and severe abnormal metabolic indicators. In clinical practice, patients with early non-response should be given a targeted management strategy, antipsychotic drugs should be switched on time, and active and effective interventions for their metabolic disorders should be given.

## Introduction

The metabolic syndrome is known to be one of the most common comorbidities of schizophrenia that lacks effective preventive measures, and they are intricately intertwined [1, 2]. It is commonly believed that prescribing atypical antipsychotics to patients with mental illness is a high risk of developing metabolic syndromes, such as weight gain, abnormal glucose metabolism, and abnormal lipid metabolism [3, 4]. However, people with schizophrenia may develop metabolic disorders earlier than prescribed antipsychotics. Some studies have even found that metabolic abnormalities such as insulin resistance and impaired glucose tolerance are already present in schizophrenia patients before antipsychotic drugs are prescribed [5–7]. According to reports, approximately 60% of patients with schizophrenia also meet the diagnostic criteria for metabolic syndrome in the United States, whereas the proportion of metabolic syndrome in the general population is only 30% [8]. Another meta-analysis found that patients with schizophrenia had a higher prevalence of metabolic disorders, obesity, type 2 diabetes, and hypercholesterolemia, estimated to be 3–5 times higher than in the general population [9]. There are also some indisputable facts: schizophrenia is associated with a significant reduction in lifespan [10] and that metabolic disorders play a unique and important role in premature death [11]. In other words, the most immediate reality following antipsychotic prescription is that while antipsychotics are recognized as irreplaceable in improving patients' psychiatric symptoms, their role in curbing and mitigating the risk of metabolic syndrome and the health risks of the metabolic syndrome is negative and counterproductive.

Early onset hypothesis of antipsychotic drug action, confirmed and extended by *Stefan Leucht* et al., primarily implies that patients taking antipsychotics improve in psychopathology more quickly in the first 1–2 weeks than 3–4 weeks and beyond [12]. Patients with a good drug response are often a strong predictor of continued use of the same drug and a good treatment effect in the first 2 weeks after drug treatment [13]. In addition, patients who did not improve their symptoms within the first 2 weeks of treatment were also less likely to respond later [14]. Researchers confirmed that early response to antipsychotic therapy was a significant predictor of subsequent response to treatment in patients with first-episode psychosis [15, 16]. In addition, similarly, patients with poor early response had significantly poorer later clinical outcomes and functional recovery [15, 17]. As a result, timely psychopathological evaluation of patients in the first 2 weeks of disease treatment, as well as timely adjustment of treatment strategies based on the evaluation results, plays a critical role in the clinical treatment and management of mental diseases, shortening hospitalization time and lowering medical burden.

A meta-analysis found that improvements in overall symptom severity were associated with increases in body weight (BW), body mass index (BMI), total cholesterol (TC), and low-density lipoprotein (LDL), and decreases in HDL levels in 18 commonly used antipsychotics [18]. Few people have systematically researched the impact of early or delayed response after antipsychotic drug treatment on short-term metabolic indicators in schizophrenia patients. This paper simulates a realistic clinical environment, randomly prescribes commonly used antipsychotic drugs for first-treatment drug-naïve schizophrenia patients and uses a retrospective analysis method to compare the difference between early response and early non-response in the short-term remission rate of patients. In combination with previous studies, we describe the changing trend of the psychopathology of patients with early response, analyze the difference in related metabolic parameters between the patients with the early response and without early response, and attempt to provide a reference for formulating targeted antipsychotic switching regimen and metabolic index control strategies.

## Methods

### Subjects

From May 2019 to September 2020, 143 patients with schizophrenia admitted to Wuhan Mental Health Center were selected for the present study.

Patients are required to meet the following inclusion criteria:

1. Meet the diagnostic criteria for schizophrenia in the International Classification of Diseases 10th Revision (ICD-10), and the total duration of the disease should not exceed 6 years.
2. No antipsychotic drugs were taken prior to the first hospitalization, or the duration of drugs did not exceed 1 week.
3. The enrolled samples were all Chinese Han people.
4. Male or female with 18–45 years.
5. The total score of the Positive and Negative Symptom Scales (PANSS) was more than 60 points, and the number of items with 4 points or more among the seven items included in the positive symptom scale is not less than 3.

Exclusion criteria: exclude lactating and pregnant women, serious somatic diseases, confirmed as diabetes, hypertension, and immune system diseases; exclude bipolar disorder, intellectual development disorder, a mental disorder caused by epilepsy, major depressive disorder, and other mental illnesses.

### Research design

This study was designed as a retrospective study. We used a self-made Excel spreadsheet in the electronic medical record system to extract demographic information and general clinical information of the enrolled patients. On the day of admission, PANSS was used to complete the evaluation of the degree of psychopathology of the patients, and the patient’s body weight (BW), abdominal circumference (AC), body mass index (BMI), etc. were measured or calculated. The next day after admission, fasting blood was drawn, routine hematology was performed. Fasting blood glucose (FBG), blood lipids, renal function and prolactin (PRL), etc., were recorded one by one in the self-made EXCLE table. The aforementioned hematological indexes were detected and re-recorded at the end of the 6th week. We divided the patients into early responder and early non-responder groups according to the PANSS score reduction rate on the second weekend after taking antipsychotics. Draw and compare the change curve of psychopathology, observe the difference in the remission rate between the two groups after 6 weeks of treatment, and compare the difference between the common metabolic indicators between the two groups.

Drug treatment methods: the choice of antipsychotic medication for all included patients will not be interfered with, and the patient’s tube bed doctor can select the type of antipsychotic medication that is optimal for the patient’s medical characteristics, and antipsychotics were increased to an effective therapeutic dose within 2 weeks. After the patient's antipsychotic medication was selected, the principles of full dose, full course and single dose were strictly observed for the next 6 weeks. Patients were automatically withdrawn from the study if there were irresistible factors that necessitated a change in the type of antipsychotic or a combination of other types of antipsychotics during the study. According to the drug response, trihexyphenidyl, propranolol, and benzodiazepines can be prescribed.

The symptomatic assessment was conducted by two psychiatrists with unified training in the Wuhan mental health center. PANSS scores were performed on the included samples on the day of admission, the 2nd weekend, the 4th weekend, and the 6th weekend. The PANSS score reduction rate (%) = (baseline score—post-treatment score)/(baseline score—30) × 100%. We defined the PANSS score reduction rate greater than or equal to 20% at the end of the 2nd week as the early response group (labeled as group A) and denied the early non-response group (labeled as group B) [19]. It is considered “remission” if the score reduction rate is greater than or equal to 75%. The PANSS is further divided into three subscales: Positive Symptoms Scale (PSS, items P1–P7), Negative Symptoms Scale (NSS, items N1–N7), and General Pathological Scale (GPS, G1–G16). The Positive symptom reduction rate (%) = (baseline score-post—treatment score)/(baseline score—7) × 100%; the Negative symptom reduction rate (%) = (baseline score—post-treatment score)/(baseline score—7) × 100%; the General Pathological Scale (%) = (baseline score—post-treatment score)/(baseline score—16) × 100%.

### Data analysis

The normally distributed continuous measurement data obtained were expressed as the mean and standard deviation, and the categorical variables as counts. Paired *t* test was used to compare the same group of data before and after, and an independent sample *t* test was used to compare the data of different groups. The Chi-square test or Fisher's precision probability was used to compare rates. Finally, to compare the differences in each metabolic parameter between the different treatment response subgroups before and after treatment, we used a 2 × 2 ANOVAs, considering the treatment response (2 levels: early response and early non-response) and the duration of treatment (2 levels: baseline and 6th week). The main effects of treatment time and subclinical group were examined, as well as the treatment time × subclinical group interaction response. The significance level of all statistical tests was set as *P* < 0.05 (two tails). Data analysis was performed using the IBM SPSS (version 26.0, SPSS Inc., Chicago, IL, USA). The figures were plotted using the GraphPad Prism software (version 8.4.3; GraphPad Software Inc., La Jolla, CA, USA).

## Results

### General clinical treatment characteristics

By the endpoint of the study, a total of 143 patients who met the criteria were enrolled in this study. They were on a single dose of each of the following seven antipsychotic drugs: aripiprazole, ziprasidone, risperidone, olanzapine, quetiapine, perphenazine, and haloperidol. They were treated with general demographics and prescribed medication, as shown in Table 1.

**Table 1:** The general clinical characteristics of the included patients

Index	Included patients (*n* = 143)
Age—years	
Mean (SD)	29.37 (7.30)
Range	18–45
Gender—(*n*, %)	
Female	80, 55.94%
Male	63, 44.06%
Onset age	24.41 (6.53)
Course of disease	2.97 (1.79)
Marital status—(*n*, %)	
Unmarried	63, 44.06%
Married	66, 46.15%
Divorced	14, 9.79%
Living condition—(*n*, %)	
With spouse	62, 43.36%
With parents	78, 54.54%
Live alone	3, 2.10%
Educational background—(*n*, %)	
Bachelor degree	7, 4.90%
College degree	8, 5.59%
Secondary specialized and high school	36, 25.17%
Junior high school	69, 48.25%
Primary school	22, 15.38%
Illiteracy	1, 0.7%
Family history	
Positive	35, 24.48%
Negative	108, 75.52%
Prescription drugs—(*n*, %)	
Aripiprazole	24, 16.78%
Olanzapine	27, 18.88%
Quetiapine	22, 15.38%
Risperidone	28, 19.58%
Ziprasidone	21, 14.69%
Perphenazine	9, 6.29%
Haloperidol	12, 8.39%

### PANSS score reduction rate and grouping after 2 weeks

The enrolled patients were divided into two groups after 2 weeks of treatment based on the PANSS score reduction rate, with 70 cases in the early response group, accounting for 48.95%, and 73 cases in the early non-response group, accounting for 51.05% (Table 2).

**Table 2:** Patient grouping based on PANSS score reduction rate after 2 weeks

	*N*, %	Baseline	2 weeks later	Reduction rate (%)
Total score	143, 100	89.15 ± 11.19	76.14 ± 14.61	22.62 ± 17.84
Group A	70, 48.95	86.94 ± 10.20	66.06 ± 10.75	37.07 ± 14.05
Group B	73, 51.05	91.27 ± 11.74	85.82 ± 10.79	8.77 ± 6.44

Group A: early response group, reduction rate ≥ 20%; Group B: early non-response group, reduction rate < 20%

### Comparison of differences in clinical parameters between the early response group and the early non-response group

There were no statistically significant differences between the two groups in demographics and the type of antipsychotic medication prescribed (Table 3).

**Table 3:** Sociodemographic and clinical characteristics between the early response group and the early non-response group

Variable	Group A	Group B	*t* ${/F/\chi }^{2}$	*P*
Age—years	28.22 ± 7.20	30.47 ± 7.27	−1.86	0.065
Gender—(*n*, %)			1.96	0.161
Female	34, 48.57%	46, 63.01%		
Male	36, 51,43%	27, 36.99%		
Onset age	23.93 ± 6.44	24.88 ± 6.62	−0.87	0.387
Course of disease	2.73 ± 1.93	3.21 ± 1.63	−1.60	0.112
Marital status—(*n*, %)			4.77	0.092
Unmarried	32, 45.71%	31, 42.47%		
Married	35, 50.00%	31, 42.47%		
Divorced	3, 4.29%	11, 15.07%		
Living condition—(*n*, %)			0.80	0.671
With spouse	32, 45.71%	30, 41.10%		
With parents	36, 51.43%	42, 57.53%		
Live alone	2, 2.86%	1, 1.37%		
Educational background—(*n*, %)			1.78	0.879
Bachelor degree	3, 4.29%	4, 5.48%		
College degree	3, 4.29%	5, 6.85%		
Secondary specialized and high school	18, 25.71%	18, 24.66%		
Junior high school	35, 50.00%	34, 46.58%		
Primary school	10, 14.29%	12, 16.44%		
Illiteracy	1, 1.43%	0, 0.00%		
Family history			0.11	0.736
Positive	18, 25.71%	17, 23.29%		
Negative	52, 74.29%	56, 76.71%		
Prescription drugs—(*n*, %)			11.83	0.066
Aripiprazole	10, 14.29%	14, 19.18%		
Olanzapine	20, 28.57%	7, 9.59%		
Quetiapine	9, 12.86%	13, 17.81%		
Risperidone	16, 22.86%	12, 16.44%		
Ziprasidone	7, 10.00%	14, 19.18%		
Perphenazine	3, 4.29%	6, 8.22%		
Haloperidol	5, 7.14%	7, 9.59%		

Group A: early response group; Group B: early non-response group

*PANSS* Positive and Negative Symptoms Scale Score, *PSS* Positive Symptom Scale Score, *NSS* Negative symptom scale score, *GPS* General Pathology Scale Score

### Changes in the scores and reduction rates of the PANSS and the three subscales

**Fig. 1:**
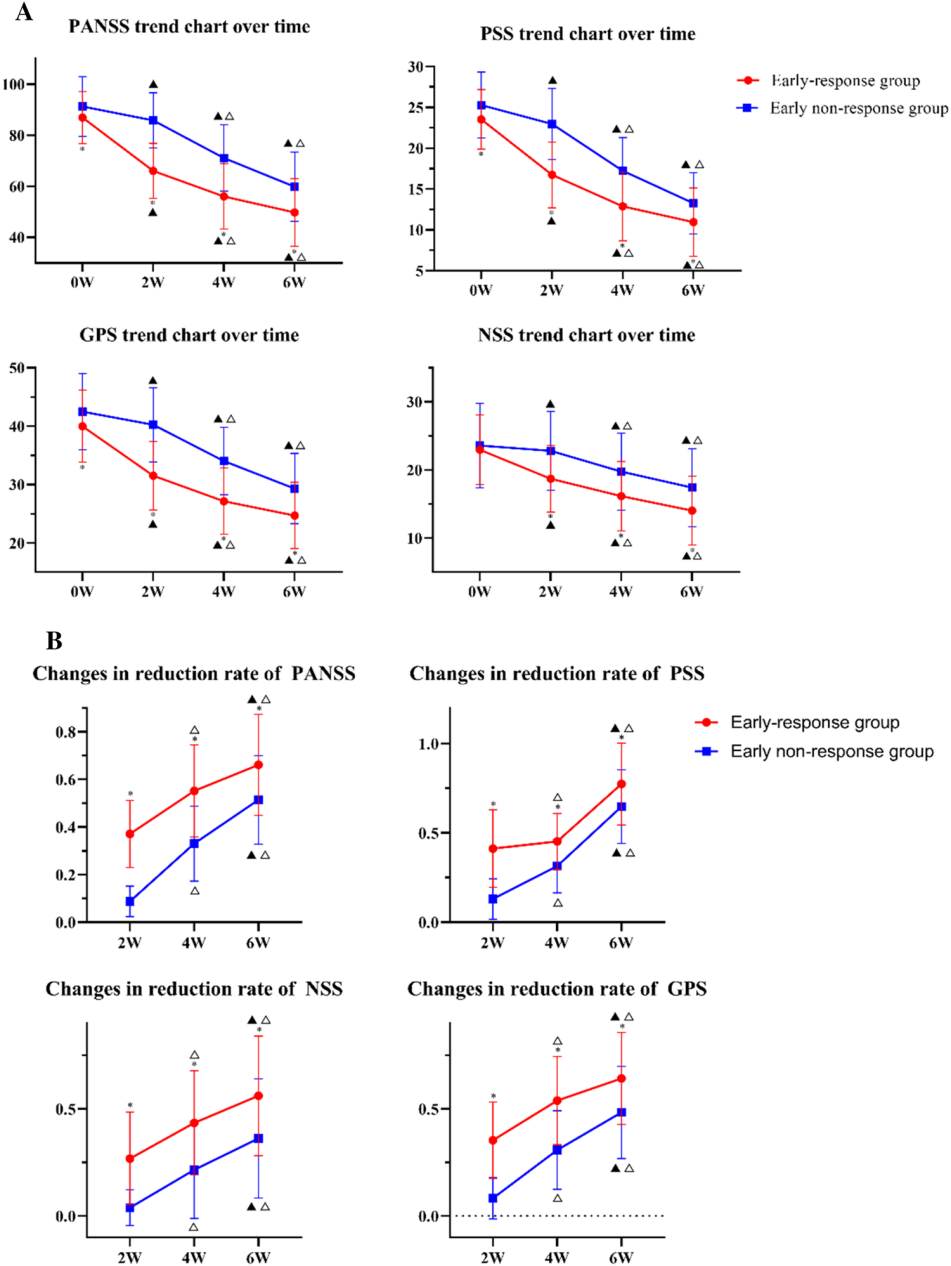
**A** Changes in PANSS and three subscale scores across groups. **B** Changes in PANSS and its three subscales’ score reduction rates. *PANSS* Positive and Negative Symptoms Scale Score, *PSS* Positive Symptom Subscale Score, *NSS* Negative Symptom Subscale score, *GPS* General Pathology Subscale Score; *P < *0.05*, compared with the early non-response group in the same period; ▲*P* < *0.05*, compared to baseline (the first set of data of each image is defined as baseline data); △*P* < *0.05*, compared to 2 weeks ago

Compared with the early non-responder group, the PANSS total score, PSS score, and GPS score in the early response group were significantly lower at the baseline, 2nd week, 4th week, and 6th week, and the differences were statistically significant (*P* = *0.020*, *P* < *0.001*, *P* < *0.001*, *P* < *0.001*, respectively), (*P* = *0.007*,* P* < *0.001*,* P* < *0.001*,* P* = *0.001*, respectively), (*P* = *0.020*, *P* < *0.001*, *P* < *0.001*, *P* < *0.001*, respectively). The NSS scores at 2 weeks, 4 weeks, and 6 weeks were significantly lower than those in the early non-response group, and the difference was statistically significant (*P* = *0.020*, *P* < *0.001*, *P* < *0.001*, respectively) (Fig. 1A). Compared with the early non-response group, the reduction rate of PANSS, PSS, PNS, and GPS in the early response group were significantly increased in the 2nd week, 4th week, and 6th week and the differences were statistically significant (*P* < *0.001*, *P* < *0.001*, *P* < *0.001* respectively), (*P* < *0.001*, *P* < *0.001*, *P* = *0.001*, respectively), (*P* < *0.001*, *P* < *0.001*, *P* < *0.001*, respectively) (Fig. 1B).

### Comparison of remission rates among different groups at study endpoints

**Fig. 2:**
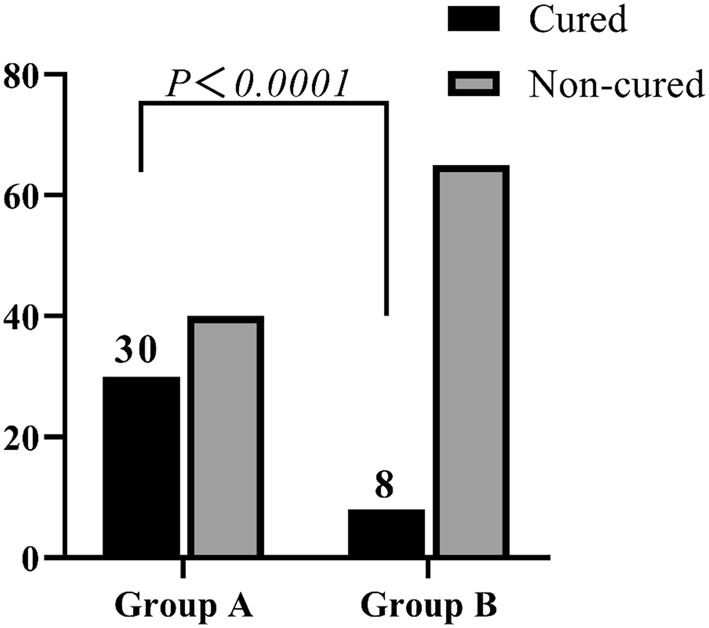
Comparison of differences in remission rates at study endpoints. Group A: early response group; Group B: early non-response group

At the 6th week, the number of remission cases in the early response group was 30, accounting for 42.86%; and 8 remission cases, accounting for 10.96% in the early non-response group. There was a statistically significant difference in the proportion of remission patients between the two groups (*P* < *0.001*) (Fig. 2).

### Comparison of differences in metabolism-related parameters at study endpoints

At the 6th week, the BW, BMI, blood creatinine (BC), blood uric acid (UA), total cholesterol (TC), triglyceride (TG), low-density lipoprotein (LDL), FBG, PRL of the enrolled samples were significantly increased, with a significance level (*P*) of *0.013*, *0.011*, < *0.001*, *0.010*, < *0.001*, < *0.001*, *0.002*, < *0.001*, and < *0.001*, respectively. In contrast, high-density lipoprotein (HDL) was significantly decreased (*P* = *0.002*) (Table 4). ANOVAs revealed a significant effect of treatment time on BC, UA,TC,TG,HDL,LDL,FBG,PRL (*F* = *22.84, P* < *0.001; F* = *4.13, P* = *0.043; F* = *57.84, P* < *0.001; F* = *78.42, P* < *0.001; F* = *64.98, P* < *0.001; F* = *10.67,P* = *0.001; F* = *74.58, P* < *0.001; F* = *30.77, P* < *0.001*; respectively), a significant negative effect of early non-response to treatment on AC,BC,TG,FBG (*F* = *6.84, P* = *0.009; F* = *7.88, P* = *0.005; F* = *16.39, P* < *0.001*; respectively) and a significant effect of time × subgroup interaction on TG (*F* = *10.16, P* = *0.002*) (Table 5).

**Table 4:** Comparison of common metabolic parameters before and after treatment

	Baseline	6th week	*t*	*P*
BW—kg	58.90 ± 11.18	59.49 ± 10.08	−2.53	0.013*
BMI—kg/m^2^	21.65 ± 3.33	21.93 ± 3.49	−2.59	0.011*
AC—cm	78.56 ± 9.29	78.75 ± 9.51	−0.98	0.328
BUN—mmol/L	3.74 ± 1.20	3.67 ± 1.30	0.49	0.624
BC—mmol/L	57.23 ± 12.90	66.90 ± 20.81	−4.54	< 0.001*
UA—mmol/L	387.06 ± 115.10	415.1 ± 116.66	−2.60	0.010*
TC—mmol/L	3.84 ± 0.75	4.70 ± 1.13	−7.72	< 0.001*
TG—mmol/L	1.10 ± 0.58	2.43 ± 1.73	−9.27	< 0.001*
HDL—mmol/L	1.18 ± 0.24	0.95 ± 0.33	8.100	< 0.001*
LDL—mmol/L	2.18 ± 0.61	2.46 ± 0.84	−3.22	0.002*
FBG—mmol/L	4.52 ± 0.77	5.66 ± 1.42	−8.53	< 0.001*
PRL—ng/mL	18.04 ± 13.49	37.06 ± 40.05	−5.89	< 0.001*

*BW* body weight, *BMI* body mass index, *AC* abdominal circumference, *BUN* blood urea nitrogen, *BC* blood creatinine, *UA* blood uric acid, *TC* total cholesterol, *TG* triglyceride, *HDL* high-density lipoprotein, *LDL* low-density lipoprotein, *FBG* fasting blood glucose, *PRL* prolactin; *P < *0.05*

**Table 5:** Common metabolic parameters before and after treatment in patients with schizophrenia, Grouped by early treatment response or not

Parameters	Group A (*n* = 70)		Group B (*n* = 73)		Time *F* (*P* value)	Subgroup *F* (*P* value)	Time × subgroup *F* (*P* value)
	Baseline	6th week	Baseline	6th week			
BW—kg	60.39 ± 11.05	60.71 ± 10.62	57.48 ± 11.20	58.32 ± 11.46	0.20 (0.659)	4.08 (0.044*)	0.04 (0.841)
BMI—kg/m^2^	21.80 ± 3.23	22.08 ± 3.24	21.50 ± 3.45	21.78 ± 3.72	0.48 (0.489)	0.54 (0.463)	0.00 (0.986)
AC—cm	80.07 ± 8.58	80.54 ± 8.84	77.11 ± 9.76	84.03 ± 9.87	0.03 (0.858)	8.67 (0.004*)	0.06 (0.803)
BUN—mmol/L	3.80 ± 1.21	3.68 ± 1.20	3.73 ± 1.32	3.61 ± 1.28	0.27 (0.607)	0.68 (0.409)	0.00 (0.997)
BC—mmol/L	59.22 ± 12.38	70.31 ± 21.82	63.61 ± 19.38	70.33 ± 21.83	22.84 (< 0.001*)	6.84 (0.009*)	0.49 (0.486)
UA—mmol/L	383.27 ± 106.46	390.70 ± 123.44	396.17 ± 107.91	433.32 ± 122.47	4.13 (0.043*)	2.66 (0.104)	1.18 (0.278)
TC—mmol/L	3.83 ± 0.73	4.76 ± 1.13	3.84 ± 0.77	4.64 ± 1.13	57.84 (< 0.001*)	0.20 (0.656)	0.34 (0.560)
TG—mmol/L	1.13 ± 0.54	1.97 ± 1.36	1.07 ± 0.61	2.86 ± 1.94	78.42 (< 0.001*)	7.88 (0.005*)	10.16 (0.002*)
HDL—mmol/L	1.18 ± 0.25	0.98 ± 0.28	1.18 ± 0.23	0.93 ± 0.20	64.98 (< 0.001*)	1.06 (0.305)	0.70 (0.404)
LDL—mmol/L	2.15 ± 0.59	2.57 ± 0.83	2.22 ± 0.62	2.56 ± 0.84	10.67 (0.001*)	0.80 (0.371)	2.67 (0.103)
FBG—mmol/L	4.32 ± 0.68	5.31 ± 1.04	4.41 ± 1.04	5.99 ± 1.65	74.58 (< 0.001*)	16.39 (< 0.001*)	1.24 (0.266)
PRL—ng/mL	19.08 ± 14.78	37.92 ± 37.79	18.31 ± 12.14	38.37 ± 25.08	30.77 (< 0.001*)	2.74 (0.099)	0.90 (0.344)

Group A: early response group; Group B: early non-response group

*BW* body weight, *BMI* body mass index, *AC* abdominal circumference, *BUN* blood urea nitrogen, *BC* blood creatinine, *UA* blood uric acid, *TC* total cholesterol, *TG* triglyceride, *HDL* high-density lipoprotein, *LDL* low-density lipoprotein, *FBG* fasting blood glucose, *PRL* Prolactin; *P < *0.05*

## Discussion

In this study, we described the change curve of the psychopathology of early responders and early non-responders. We observed the changes in major metabolic indexes of patient groups with different responses in the early stage of treatment over time according to the prescription of seven commonly used antipsychotics for first-treatment drug-naïve schizophrenia patients. Our main findings are as follows: 1. Early non-responders made up about half of our sample population (51.05%). 2. Psychopathology in early responders was relatively mild than early non-responders, including positive symptoms and general pathological symptoms and the magnitude of change in responders’ psychiatric symptoms (referring to the reduction rate) was more significant. 3. At the end of the study, the absolute number of early responders who met the remission criteria also had an absolute advantage over those with poor early response. 4. Whether the early response is good or bad, the deterioration trend of metabolic indicators is widespread and noticeable. However, it must be emphasized that, in the short term, abnormalities in metabolic markers in schizophrenia treated with antipsychotics were characterized by broad time-cumulative effects, and these abnormalities were more severe in early non-response patients.

There is still a lot of evidence that early response to psychiatric illness is a strong predictor of later efficacy and functional recovery [20–22], particularly in the second week of efficacy response [23]. The present study also supports the above conclusion. During the 6-week observation period, 42.86% of the early response patients were in remission, while 10.96% of early non-response patients. In clinical practice, 6 weeks is generally considered the period for acute phase treatment and is the maximum time threshold for psychiatrists to decide whether to switch the type of drug prescribed for a patient who does not respond well to treatment. According to the present findings, 89.04% of patients with poor early response to treatment would not achieve better outcomes within 6 weeks if they continued to take the same drug. A recent multi-center clinical study found that timely switching of the drug could significantly improve symptoms in schizophrenia patients whose symptoms did not improve significantly after 2 weeks of adequate olanzapine or amisulpride treatment compared with continuing the original drug treatment, and the proportion of patients who achieved symptom relief criteria increased [24]. This finding also further supports the confidence level of the recommendations based on the present research. Therefore, we suggest that patients with poor early response to treatment can reduce the time threshold for switching types of antipsychotic drugs to 2 weeks after treatment to minimize unnecessary hospitalization cycles and medical costs.

It is not difficult to find that the comorbidity of mental illness with metabolic disorders is prevalent. According to a large-scale clinical study, up to 60.7% of patients with first-episode drug-naïve schizophrenia have at least one of the five components of metabolic syndrome, compared with only 36.5% in the general population [25]. In other words, schizophrenia itself is a risk factor for metabolic disorders [26]. Furthermore, it is crucial and well-studied that the effect of antipsychotic drugs on patients' metabolic abnormalities is both evident and negative, and the underlying mechanism is also quite complex [27]. As far as inpatients are concerned, apart from the disease and drug factors, it should also include changes in the patient's living habits and environment. For example, due to space and equipment limitations in psychiatric hospitals, it is difficult for patients to control abnormalities in metabolic indicators through exercise and fitness. In short, schizophrenia with the comorbid metabolic disorder is a complex result of interweaving and interaction of many factors. Although we included a broader range of antipsychotic drug classes to weaken the effect of antipsychotic drug type on metabolism and to more objectively model the real-world environment in which Chinese patients are prescribed antipsychotics, the present study also reported that abnormal metabolic indicators after treatment were common and numerous in the included patients.

Known studies remain inconsistent on the relationship between various metabolic markers and psychopathology. A longitudinal cohort study of first-episode schizophrenia found no correlation between psychopathology polygenic scores and metabolic progression [28]. Another meta-analysis reported that improvements in psychopathology were associated with metabolic disturbances [18]. The present study found that regardless of early response to treatment, enrolled patients had elevated levels of a wide range of metabolic parameters (HDL levels are significantly reduced, but HDL is a protective lipoprotein, and its reduced levels also have a negative impact on the body [29, 30]). Some metabolic indicators were higher in patients with poor early response to treatment than in patients with good early response to treatment, as found at the end of this study. Thus, patients who do not respond early may be more negatively affected by metabolic disorders in the short term. Although this cannot be ruled out that for patients with poor response to treatment, physicians will be more active in implementing a regimen of increasing the dose of antipsychotic drugs within the scope of the rules [31]. *Xiaoe Lang* et al. also found that some metabolic indicators (e.g., WC, FBG) were positively correlated with psychopathology scores in drug-naïve patients in their first episode of schizophrenia [32]. In our study, the early non-response group maintained more severe psychopathological symptoms throughout the study period compared to the early response group. This may also be another important reason why many metabolic indicators were worse in the early non-response group. More critically, however, a clear association between premature death and metabolic syndrome has been found in patients with schizophrenia [33]. Therefore, no matter what causes the metabolic disorder to be more serious, we necessitate paying special attention to those who do not respond well to treatment early on, as well as providing interventions in a planned and purposeful manner to reduce the risk of metabolic syndrome.

In conclusion, we found that those with early response to treatment had a higher short-term remission rate; post-treatment metabolic disorders were common and widespread regardless of early response or not. However, the abnormal metabolic indicators were more widespread and severe in the early non-responders. In clinical practice, patients with poor early response should be given a targeted management strategy, antipsychotic drugs should be switched on time, and active and effective interventions for their metabolic disorders should be given.

## Data Availability

The data sets used and/or analysed during the current study available from the corresponding author on reasonable request.

## Abbreviations

PANSS: Positive and negative symptoms scale score
PSS: Positive symptom scale score
NSS: Negative symptom scale score
GPS: General pathology scale score
BW: Body weight
BMI: Body mass index
AC: Abdominal circumference
BUN: Blood urea nitrogen
BC: Blood creatinine
UA: Blood uric acid
TC: Total cholesterol
TG: Triglyceride
HDL: High-density lipoprotein
LDL: Low-density lipoprotein
FBG: Fasting blood glucose
PRL: Prolactin
ICD-10: The International Classification of Diseases 10th Revision
